# Recent Advances in Bioinspired Gel Surfaces with Superwettability and Special Adhesion

**DOI:** 10.1002/advs.201900996

**Published:** 2019-07-22

**Authors:** Pengchao Zhang, Chuangqi Zhao, Tianyi Zhao, Mingjie Liu, Lei Jiang

**Affiliations:** ^1^ Key Laboratory of Bioinspired Smart Interfacial Science and Technology of Ministry of Education School of Chemistry Beihang University Beijing 100191 P. R. China; ^2^ International Research Institute for Multidisciplinary Science and Beijing Advanced Innovation Center for Biomedical Engineering Beihang University Beijing 100191 P. R. China

**Keywords:** adhesion, hydrogels, liquid‐like surfaces, organogels, superwettability

## Abstract

Engineering surface wettability is of great importance in academic research and practical applications. The exploration of hydrogel‐based natural surfaces with superior properties has revealed new design principles of surface superwettability. Gels are composed of a cross‐linked polymer network that traps numerous solvents through weak interactions. The natural fluidity of the trapped solvents confers the liquid‐like property to gel surfaces, making them significantly different from solid surfaces. Bioinspired gel surfaces have shown promising applications in diverse fields. This work aims to summarize the fundamental understanding and emerging applications of bioinspired gel surfaces with superwettability and special adhesion. First, several typical hydrogel‐based natural surfaces with superwettability and special adhesion are briefly introduced, followed by highlighting the unique properties and design principles of gel‐based surfaces. Then, the superwettability and emerging applications of bioinspired gel surfaces, including liquid/liquid separation, antiadhesion of organisms and solids, and fabrication of thin polymer films, are presented in detail. Finally, an outlook on the future development of these novel gel surfaces is also provided.

## 1 Introduction

Natural evolution and election has led to the development of numerous mysterious living organisms endowed exquisite surfaces with unique properties, especially superwettability.^1^, ^2^, ^3^, ^4^, ^5^ As the first and well‐known example, lotus leaf has been widely studied because of its self‐cleaning feature, which has been demonstrated to be attributed to its superhydrophobicity and low water adhesion in air.^6^, ^7^ To date, extensive studies on biological surfaces, such as rose petals,^8^ rice leaves,^6^ and gecko feet,^9^ have revealed that the superwettability arises from the synergy of the surface chemical composition and multiscale structures. Following the above principle, scientists have established a superwettability system, motivating the explosive development of functional solid surfaces with exceptional functions for extraordinary applications from agriculture and industry to our daily life.^4^, ^10^, ^11^, ^12^, ^13^, ^14^ In recent decades, solid surfaces with superwettability have been a focus of research. Although significant progress has been made, solid surfaces still present some severe limitations in practical applications. For example, solid surfaces, which are usually rigid, are rarely used when there is a need for soft and deformable properties. Additionally, solid surfaces suffer from the loss of superwettability due to the irreversible damage of their surface micro/nanostructures or surface chemical compositions under severe conditions.^15^ Thus, new design principles and engineering strategies are urgently needed to develop artificial surfaces with superwettability and special adhesion to address the above challenges.

Organisms living in their natural environments, such as fishes,^16^ seaweed,^17^ and tree frogs,^18^ employ soft organic materials, especially hydrogels, which mainly consist of proteins and polysaccharides, as their surficial materials. In contrast to solid materials, hydrogels are composed of a water phase entrapped in a three‐dimensional (3D) cross‐linked polymer network through weak interactions (for example, hydrogen bonds and van der Waals forces).^19^, ^20^, ^21^ Accordingly, hydrogels behave like a solid that can maintain their shapes. Moreover, hydrogels are wet and soft and can be considered to be in a quasi‐liquid phase, in stark contrast to solid materials, which are usually dry and hard. The liquid phase trapped inside the cross‐linked polymer networks can maintain their mobility to some extent, causing the hydrogels to have liquid‐like surfaces. This unique property makes gel surfaces excellent candidates for the development of new artificial surfaces with superwettability and special adhesion to address the issues encountered by solid surfaces. As expected, bioinspired gel surfaces have emerged as promising materials in diverse fields. For example, hydrogel surfaces inspired by fish scales show underwater superoleophobicity and have been used for highly efficient water/oil separation.^22^ Organogel surfaces inspired by the *Nepenthes* pitcher have extremely low friction with immiscible liquids, organisms, and solids, showing promising applications as self‐cleaning coatings.^23^

In this review, we summarize recent progress in bioinspired gel surfaces with superwettability and their emerging applications. First, we will introduce several representative hydrogel‐based natural surfaces with superwettability and special adhesion, and then highlight the unique liquid‐like properties, design principles, and fabrication strategies of bioinspired gel surfaces. Subsequently, several established types of gel surfaces with superwettability will be discussed in detail. Next, the emerging applications of bioinspired gel surfaces, including liquid/liquid separation, antibiofouling, antisolid adhesion, antifriction, and fabrication of functional thin polymer films, will be introduced in detail. Finally, we conclude this review by presenting the challenges and perspectives related to these functional gel surfaces.

## 2 Hydrogel‐Based Natural Surfaces with Superwettability and Special Adhesion

Numerous naturally living organisms exhibit functional surfaces with superwettability and special adhesion. Many of them have hydrogel‐based surfaces, such as fishes,^16^ seaweed,^17^ and tree frogs,^18^ and have been explored to unveil the underlying mechanisms. This section will highlight the distinctive features of several typical natural surfaces.

### 2.1 Carp Scales with Underwater Superoleophobicity

**Figure 1:**
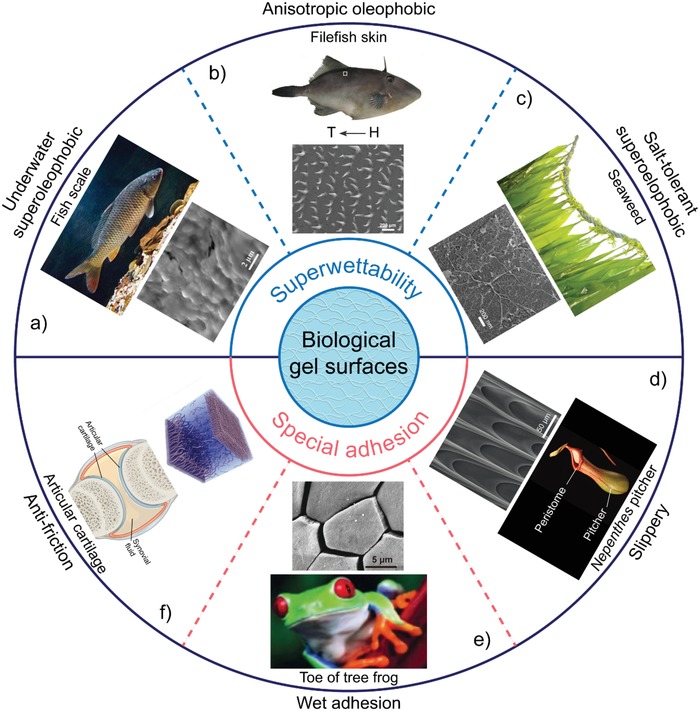
Representative biological hydrogel surfaces with superwettability and special adhesion. a) Carp scales demonstrate low adhesive, underwater superoleophobic, and self‐cleaning properties, owing to the hydrogel‐coated scales with micro/nanostructures. Reproduced with permission.^16^ Copyright 2009, John Wiley & Sons, Inc. b) Filefish skins exhibit anisotropic oleophobicity under water attributed to the oriented hook‐like spines arrayed on their skins. Reproduced with permission.^24^ Copyright 2014, John Wiley & Sons, Inc. c) Seaweeds possess stable superoleophobicity in highly saline conditions because their alginate gel covered surfaces can bond water molecules regardless of high salinity levels. Reproduced with permission.^17^ Copyright 2015, John Wiley & Sons, Inc. d) *Nepenthes* pitcher plants can capture prey with the peristome, an anisotropic surface that is completely wetted by secreted nectar and rainwater. Reproduced with permission.^26^ Copyright 2016, Nature Publishing Group. e) Tree frogs can cling to smooth surfaces using watery mucus wetted toe pads, which are composed of peg‐studded hexagonal cells. Reproduced with permission.^1^ Copyright 2017, Nature Publishing Group. f) Articular cartilage permits virtually frictionless mechanical motion within joints even under high compression due to the proteoglycan‐based hydrogels. Top: Reproduced with permission.^171^ Copyright 2017, John Wiley & Sons, Inc. Bottom: Adapted from attribution. OpenStax College [CC BY 3.0 (https://creativecommons.org/licenses/by/3.0)].

Underwater creatures are excellent examples to study underwater superwettability because of their natural living environment. The first well‐studied example is the carp with underwater superoleophobic and self‐cleaning scales, which can keep clean in oil‐polluted areas (**Figure** **1**a).^16^ Carps have densely arranged fan‐shaped scales with diameters of 4–5 mm, on which are oriented micro/nanoscaled papillae with a length of 100–300 µm and width of 30–40 µm. Such scales are usually covered by a thin layer of hydrogel‐like mucus with a strong affinity for water molecules. The multiscale structures and hydrogel‐like mucus can trap a water layer on their surfaces, resulting in the formation of a liquid‐like surface with superoleophilicity in air and superoleophobicity underwater (contact angle (CA) of 156.4° ± 3.0° for oil droplets).

The theoretical models used for the explanation of the wettability of solid surfaces in air can be utilized in the above oil/water/gel three‐phase system. Accordingly, Equation (1) can be obtained from Young' equation^16^

(1) $$\text{cos}\theta_{3}\;\;=\;\;\frac{\gamma_{l1-g}\text{cos}\theta_{1}-\gamma_{l2-g}\text{cos}\theta_{2}}{\gamma_{l1-l2}}$$

where γ_l1–g_, γ_l2–g_, and γ_l1–l2_ are the interface tension of liquid 1/air, liquid 2/air, and liquid 1/liquid 2, respectively. θ_1_, θ_2,_ and θ_3_ are the CA of liquid 1 in air, liquid 2 in air, and liquid 1 in liquid 2, respectively. When an oil droplet is deposited onto a hydrogel surface underwater, liquids 1 and liquid 2 are oil and water, respectively. Through calculation, hydrogel surfaces become oleophobic underwater. When introducing rough surface structures into a hydrogel surface, water molecules can be trapped in the rough surface structures, leading to formation of the Cassie state in the oil/water/gel system. In this case, Cassie' equation can be expressed^16^

(2) $$\text{cos}{\theta^{\prime }}_{3}\;\;=\;\;f\;\text{cos}\theta_{3}\;+\;f-1$$

where *f* is the area fraction of gels, and θ′_3_ is the CA of an oil droplet on a rough gel surface underwater. The theoretical calculations fit the experimental results very well. This innovative work demonstrated, for the first time, the possibility of studying the superwettability of materials' surfaces in the liquid phases, with promising potential for many applications, such as marine antifouling, water/oil separation, and bioadhesion.

### 2.2 Filefish Skins with Anisotropic Superoleophobicity

In contrast to carps with flaky scales, the filefish *N. septentrionalis* possesses a sandpapery bony skin covered with hook‐like spines (Figure 1b).^24^ These spines have a height of 383.7 ± 17.6 µm, width of 51.6 ± 5.4 µm, and curved tips, uniformly oriented toward the filefish tail. They are arrayed in lines with more than 100 µm between each row. This unique feature endows the skin of filefishes with robust underwater superoleophobicity (oil CA: 156.1° ± 1.8°), which is even free from disturbances caused by external forces and slight structural damage. Notably, the skin of filefishes shows anisotropic superoleophobicity underwater: oil droplets tend to roll off the surface in the head‐to‐tail direction but to pin in the opposite direction, providing a novel model for the development of anisotropic surfaces.^24^

### 2.3 Seaweed Surfaces with Salt‐Tolerant Underwater Superoleophobicity

In addition to fishes, marine plants, for example, seaweed *Saccharina japonica* (*S. japonica*), have hydrogel‐based surfaces with salt‐tolerant underwater superoleophobicity (Figure 1c).^17^ On the surface of *S. japonica*, there is a thin layer of hydrogel‐like mucilage with a rich content of polysaccharides (including carrageenan, agar, and alginate). The hydrogel‐like mucilage layer, which contains and bonds large amounts of water molecules, confers a liquid‐like property to their surfaces, leading to naturally underwater superoleophobicity (oil CA: 160.7° ± 5.0°). Importantly, such underwater superoleophobicity is insensitive to high levels of ion strength and salinity, resulting from the salt‐insensitive mucilage.

### 2.4 Slippery *Nepenthes* Pitcher

As one of the most famous carnivorous plants, the *Nepenthes* pitcher can capture insects, ants, and other small animals as their primary nutrition source (Figure 1d).^25^ It has been revealed that the capture mechanism arises from the unique surface properties of the pitcher peristome. The peristome exhibits slippery, anisotropic, and amphiphilic characteristics.^25^, ^26^ On the peristome surfaces, there are anisotropic microstructures with radial ridges composed of epidermal cells, forming a series of steps toward the internal pitcher. Additionally, the peristome surfaces can be entirely wetted by secreted nectar and rainwater, leading to the formation of homogeneous liquid‐like films on the surfaces. Under such conditions, the peristome surfaces are slippery for any external objects. Therefore, the water lubrication and anisotropic surface topography of peristome surfaces endows the *Nepenthes* pitcher with the ability to prevent the adhesion between insect feet and the pitcher: the water films disrupt attachment, whereas the surface topography leads to anisotropic friction.

### 2.5 Toes of Tree Frogs with Reversible Wet Adhesion

The tree frog (*Litoria caerulea*) can climb on smooth leaf and branch surfaces in wet environments without falling.^18^, ^27^ This reversible wet adhesion capability results from the distinctive features of their toe pads (Figure 1e).^18^ The toe pads of the tree frog are patterned with regular hexagonal epidermal cells with a diameter of ≈10 µm separated by channels with a width of ≈1 µm. The flat surface of each hexagonal epidermal cell has fine nanostructures with ≈0.1–0.4 µm diameter pegs. The toe pads are additionally entirely wetted with a watery mucus. The highly regular micro/nanostructures as well as the hydrogel‐like mucus significantly enhance the attachment forces by the close contacts and boundary friction between the epidermis and substrates, resulting in the reversible wet adhesion property.

### 2.6 Articular Cartilage with Low Friction

As an essential connective tissue, articular cartilage strikingly illustrates how hydrogel‐like materials can be utilized to achieve unique functions: it permits rapid, robust, reversible, and virtually frictionless mechanical motion within joints under stress.^28^, ^29^, ^30^ The structure of articular cartilage can be simplified into two parts: a solid‐like scaffold and a liquid‐like surface (Figure 1f). The solid part is almost incompressible due to the electrostatic repulsion interaction of proteoglycans dispersed within the cross‐linked network of collagen fibrils and is used to bear the load at low speed.^31^ The top liquid‐like surface, which is composed of collagen fibers predominantly parallel to the surfaces,^32^ can hold abundant interstitial fluid (≈75–80 wt%), providing a low‐friction and bearing load in a high‐speed situation.^33^, ^34^ Taken together, the fluid‐film and boundary lubrication in the water‐based lubricant environment lead to the low friction of articular cartilage.^30^, ^35^, ^36^

## 3 Unique Features of Gel Surfaces

**Figure 2:**
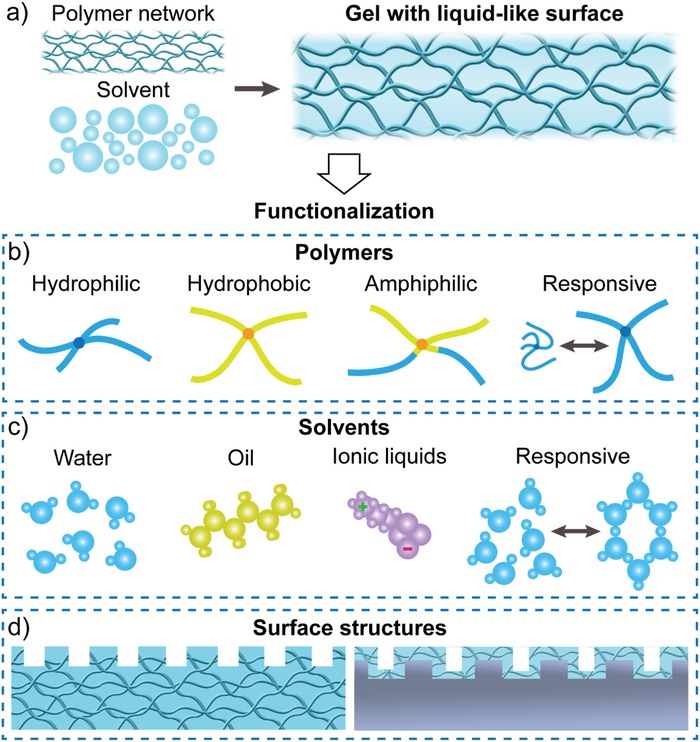
Strategies for rationally engineering gel surfaces. a) Gels are made from cross‐linked polymer networks swollen with large amounts of solvents. Thus, gel surfaces exhibit a liquid‐like state. b–d) Gel surfaces can be functionalized by engineering (b) polymers (including hydrophilic, hydrophobic, amphiphilic, and responsive polymers), (c) solvents (including water, oil, ionic liquids, and responsive solvents), and (d) surfaces structures (patterned gel surfaces or gel layers coated on structured surfaces).

Hydrogel‐like natural surfaces offer new design principle and fabrication strategies for the development of advanced surfaces with superwettability and special adhesion. As shown in **Figure** **2**a, gels are composed of a 3D cross‐linked polymer network that entraps large amounts of solvents through weak interactions (such as hydrogen bonds, van der Waals forces, and hydrophobic interaction).^19^, ^20^, ^21^ The natural fluidity of the trapped solvents confers the liquid‐like property to gel surfaces,^37^ making them significantly different from solid surfaces. Moreover, the cross‐linked polymer networks not only maintain the solid‐like shape but also endow the gel surfaces with free and dangling polymer chain ends, which play crucial roles in the adhesion and friction properties.^38^

**Figure 3:**
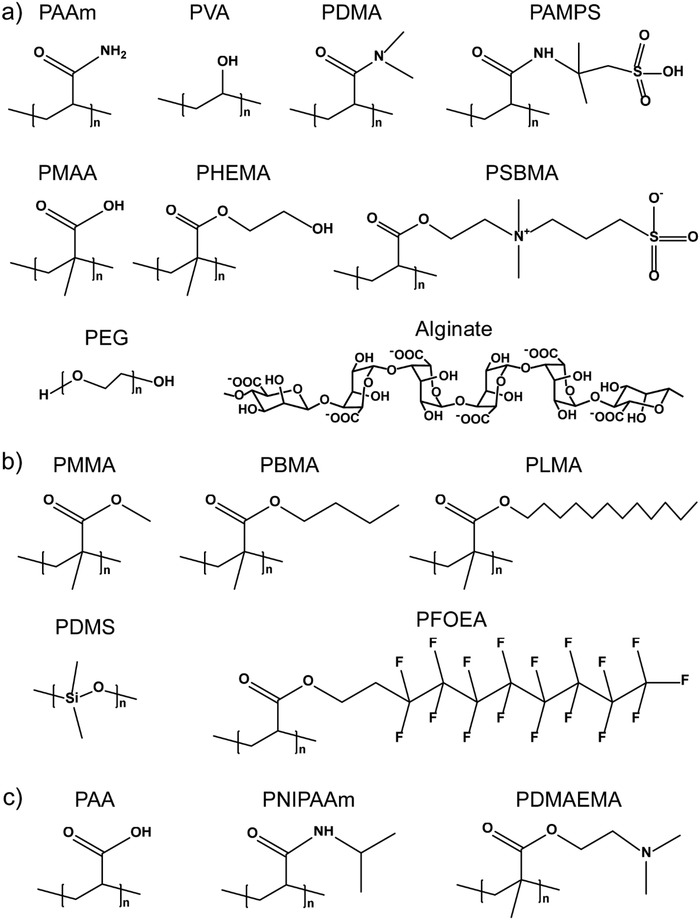
Representative polymers for engineering bioinspired gel surfaces with superwettability and special adhesion. a) Hydrophilic polymers. b) Hydrophobic polymers. c) Responsive polymers.

Until now, three strategies have been utilized to rationally engineer bioinspired gel surfaces with superwettability and special adhesion. First, the polymer networks can be ideally designed to achieve specific functions and applications. Polymers with different intrinsic wettability, such as hydrophilicity, hydrophobicity, and amphiphilicity, have been employed to fabricate gel surfaces (Figure 2b). Importantly, appropriately engineering the polymers can endow the gels with the ability to substantially and reversibly switch (e.g., swelling and shrinking) with changes in the external environment, such as temperature, pH, or ionic strength. Such an ability offers the opportunity to rationally design intelligent gel surfaces with controllable and reversible surface properties and functions. **Figure** **3** lists typical hydrophilic (Figure 3a), hydrophobic (Figure 3b), and responsive polymers (Figure 3c) that have been used to engineer bioinspired gel surfaces with superwettability and special adhesion for various applications. Second, we can also select the solvents on‐demand, for example, water, oil, ionic liquids, or responsive liquids, to achieve specific purposes (Figure 2c). Such solvents trapped in the gels can undergo a reversible transition between different states (e.g., liquid and solid‐state or high viscous and low viscous state) under the external stimulus, leading to the switching of surface properties and functions in a controllable manner. Finally, surface structures, ranging from the nanoscale to macroscale, can be employed to construct structured gel surfaces, which can be achieved by directly patterning the gel surfaces or introducing gel layers onto solid surfaces with various morphologies (Figure 2d). The development of advanced gel surfaces is highly expected with the combination of two or more of these strategies.

## 4 Engineering Bioinspired Gel Surfaces with Superwettability

**Figure 4:**
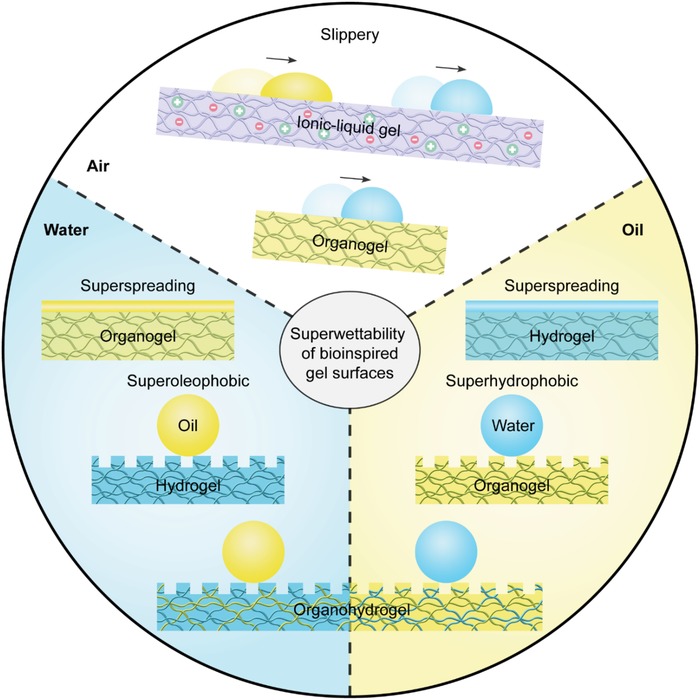
Summary of the superwettability of bioinspired gel surfaces. When depositing a liquid droplet on a gel surface, it can form a liquid/liquid interface, which has much lower friction for the movement of liquid droplets than solid surfaces. Thus, the gel surfaces exhibit an easy‐sliding property for immiscible liquids both in the air (i.e., slippery) and under the liquid phase (i.e., underwater superoleophobicity and underoil superhydrophobicity). For miscible liquids, the liquid/liquid interfaces can enhance their spreading, leading to rapid and complete spreading (i.e., superspreading).

By using the above strategies, four kinds of gel surfaces, including the hydrogel, organogel, ionic‐liquid‐gel, and organohydrogel, have been developed (**Figure** **4**). When depositing a liquid droplet on a gel surface, it can form a liquid/liquid interface, which has much lower friction for the movement of liquid droplets than a liquid/solid interface. Thus, the gel surfaces exhibit the easy‐sliding property for immiscible liquids both in air (i.e., slippery) and under a liquid phase (i.e., underwater superoleophobicity and underoil superhydrophobicity). For miscible liquids, the liquid/liquid interfaces can enhance their spreading, leading to the rapid and complete spreading (i.e., superspreading). These unique features confer special functions to the gel surfaces when encountering liquids, organisms, and solids, motivating scientists to develop novel gel surfaces and to address numerous problems in our daily life.

### 4.1 Bioinspired Hydrogel Surfaces with Underwater Superoleophobicity

**Figure 5:**
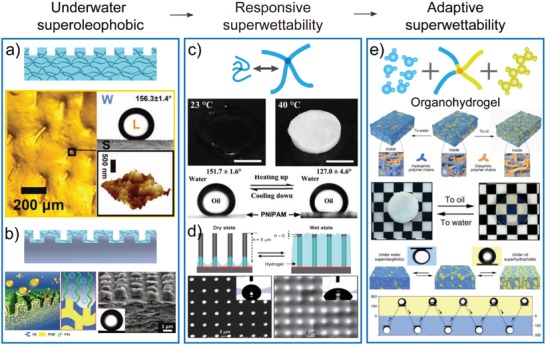
Bioinspired gel surfaces with superlyophobicity. a) Hierarchical macromolecule–nanoclay hydrogels with robust underwater superoleophobicity. Reproduced with permission.^39^ Copyright 2010, John Wiley & Sons, Inc. b) Composite hydrogel coating with robust underwater superoleophobicity, low oil‐adhesion, and excellent mechanical properties. Reproduced with permission.^43^ Copyright 2018, John Wiley & Sons, Inc. c) The thermoresponsive PNIPAAm hydrogel surface could switch the wettability from underwater superoleophobic to oleophobic when changing the environmental temperature. Reproduced with permission.^46^ Copyright 2010, Royal Society of Chemistry. d) Water‐responsive hybrid hydrogel surfaces. Reproduced with permission.^54^ Copyright 2008, Royal Society of Chemistry. e) Organohydrogel surfaces with adaptive superwettability. The organohydrogels can switch between hydrogel‐ and organogel‐like states in response to surrounding liquids and show superoleophobic under water and superhydrophobic under oil. Reproduced with permission.^56^ Copyright 2017, Nature Publishing Group.

Bioinspired hydrogel surfaces with underwater superoleophobicity have been intensively studied to mimic the functions of fish scales. For instance, Lin et al. developed a hybrid poly(*N*‐isopropylacrylamide)–nanoclay (PNIPAAm–nanoclay) hydrogel with hierarchical surface structures, exhibiting robust underwater superoleophobicity and low oil‐adhesion (**Figure** **5**a).^39^ The PNIPAAm–nanoclay hydrogels were fabricated by photoinitiated radical polymerization of the mixture of NIPAAm and clay nanoparticles. As a physical cross‐linker, clay nanoparticles can significantly enhance the mechanical strength of the prepared PNIPAAm–nanoclay hydrogels. The interaction of the rigid nanoclay and flexible polymer networks supports the stability of trapped water on the hydrogel surface and contributes to the robust superoleophobicity and low adhesion properties. Our group also developed a nonswellable poly(2‐hydroxyethyl methacrylate) (PHEMA) hydrogel with a micro/nanostructured surface, which showed superior oil‐repellency even after immersion in seawater for one‐month.^40^ Using the same hydrogels, Arora et al. fabricated a superoleophobic hydrogel surface, on which a micropattern was introduced to enhance oleophobicity.^41^, ^42^

Similarly, to mimic the hydrogel‐like mucus layer of *S. japonica*, Cai et al. fabricated calcium alginate (Ca‐alginate) hydrogel coating with salt‐tolerant oil‐repellent features on glass slides through the electrostatic interactions of polyanions and polycations.^17^ The Ca‐alginate hydrogel coating showed excellent underwater superoleophobicity (crude oil CA: 158.1° ± 2.3°) and ultralow oil‐adhesion. These properties could be retained even after a 30‐day immersion in artificial seawater. Due to its low cost and broad applicability on solid surfaces of different shapes and substrate materials, the salt‐tolerant superoleophobic Ca‐alginate hydrogel coating will advance the development of commercial oil‐repellent materials.

To further improve the mechanical performance of oil‐repellent hydrogel surfaces, Meng et al. fabricated a mechanically robust and underwater oil‐repellent surface by combining a hydrophilic PAA hydrogel coating with a layered poly(acrylic acid)/poly(vinylidene fluoride)–graphene nanosheet (PAA/PVDF–GN) interior matrix with columnar hierarchical micro/nanostructures (Figure 5b). Such surfaces exhibit underwater superoleophobicity (oil CA > 150°) and low oil adhesion (<2.8 µN) while displaying excellent mechanical properties (tensile stress > 80 MPa), even after being immersed in seawater and extremes of pH for one month.^43^

### 4.2 Bioinspired Hydrogel Surfaces with Responsive Superwettability

The designability of polymer networks endows the gel surfaces with controllable and reversible responsiveness to external stimuli, such as humidity, temperature, pH, or mechanical forces.^44^, ^45^ Under these stimuli, the polymer networks can switch their physical and chemical properties, resulting in alterations of the surface properties of gel surfaces. By using the above feature of polymers, extensive attention has been paid to control of the superwettability of gel surfaces. As the most famous case, PNIPAAm has been utilized to fabricate hydrogel surfaces with reversible wettability and adhesion to oil (Figure 5c).^46^ When the temperature is below the lower critical solution temperature (LCST) of PNIPAAm (e.g., 23 °C), the PNIPAAm hydrogels exhibit a swelling state because of the formation of intermolecular hydrogen bonds between PNIPAAm networks and water molecules. In this case, the PNIPAAm hydrogels exhibit transparent, underwater superoleophobic, and low oil‐adhesion properties. By contrast, at 40 °C (above LCST), the PNIPAAm hydrogel surfaces become opaque (shrink state): the oil droplets firmly adhere, even when turning down the hydrogel surfaces, due to the formed intramolecular hydrogen bonds between PNIPAAm chains.

The poly(dimethylamino) ethyl methacrylate (PDMAEMA) hydrogel was coated onto a stainless steel mesh to achieve the pH‐controlled transition of superwettability.^47^ The PDMAEMA hydrogel‐coated mesh was superhydrophilic in air and superoleophobic underwater for various oils. After modification with stearic acid on the PDMAEMA hydrogel surface through electrostatic attraction, the PDMAEMA hydrogel‐coated mesh became superhydrophobic and superoleophilic in air. This wettability transition could be switched by treating the PDMAEMA hydrogel‐coated mesh with an aqueous solution of 1 m NaOH for 5 min. The pH‐triggered wettability alteration between hydrophilic and superhydrophilic states was achieved on the surfaces of a vertically aligned carbon nanotube (VACNT) array functionalized with a poly(methacrylic acid‐*co*‐ethylene glycol diacrylate) (P(MAA‐*co*‐EGDA)) hydrogel.^48^ The hybrid hydrogel–VACNT surfaces were fabricated by coating the P(MAA‐*co*‐EGDA) hydrogel (50 nm thickness)on the surfaces of carbon nanotubes. Due to the pH‐responsiveness of the P(MAA‐*co*‐EGDA) hydrogel, the hybrid hydrogel–VACNT surfaces exhibited superhydrophilicity at pH 7 and hydrophilicity at pH 2. This response resulted from the ionization of carboxyl groups, which induced swelling of the P(MAA‐*co*‐EGDA) hydrogel. A similar strategy was used to fabricate a pH‐responsive surface by coating a poly(4‐vinyl pyridine) (PVP) hydrogel thin film on a silicon substrate.^49^ The PVP hydrogel showed severe and reversible tenfold swelling when switching the pH from neutral to acidic, leading to the sharp transition of surface wettability from the hydrophobic (CA: 70°) to the hydrophilic state (CA: 20°).

In an indirect and undamaged manner, light has also been utilized to manipulate the surface wettability of photoresponsive hydrogels. For instance, Joseph et al. described a photo‐/thermoresponsive surface by covalently grafting a thin layer of spirobenzopyran (SP) functionalized PNIPAAm hydrogel on the nanostructured olefin substrates.^50^ Upon light irradiation or temperature changes, such surfaces exhibited a transition from superhydrophilicity and moderate hydrophobicity with water CAs of 5° and 123.1°, respectively. The light‐induced reversible wettability switching can be attributed to the synergistic effect of the photoisomerization of SP and the hydration/dehydration of the PNIPAAm chain. Moreover, a superhydrophobic hydrogel surface with photoresponsive superficial layer was fabricated using the confined quaternization reaction at the hydrogel/oil interface to covalently tether the spiropyran derivative (IBSP) on the hydrogel surface.^51^ Due to the photoresponsive ring open and close mechanism of IBSP, the reversible switch of underwater oil wettability and oil adhesion on the IBSP‐modified hydrogel was achieved by alternately UV and visible light illumination.

Recently, a general strategy was developed to fabricate programmable responsive hydrogel surfaces that could reversibly transit from the superhydrophobic to the superhydrophilic state under a series of external stimuli. The fabrication of programmable responsive hydrogel surfaces involved the coating of silanized micrometer glass particles into a stimuli‐responsive hydrogel.^52^ For example, when embedding the silanized particles into stretch‐responsive hydrogel surfaces, the formed composite surfaces were superhydrophobic becoming superhydrophilic when stretching the surfaces to 600%. Using the same method, the authors further fabricated pH‐responsive as well as temperature‐responsive composite hydrogel surfaces. These composite hydrogel surfaces can be used as stimuli‐responsive gates to control their chemical permeability by applying external stimuli.

Control of the swelling state of hydrogels offers another simple way to achieve the switch of the wettability and adhesion properties of hydrogel surfaces. Sidorenko and co‐workers presented a hydrogel‐supported nanostructured surface and demonstrated its application in controlled reversible switches of surface wettability (Figure 5d).^53^, ^54^, ^55^ The hydrogel‐supported nanostructured surface was fabricated by infusing polyacrylamide (PAAm) hydrogel into the interspace of nanowire arrays on silicon surfaces, which were originally superhydrophobic. When exposed to water, the dry hydrogel could swell and flood the nanostructures, leading to alteration of the surface wettability from superhydrophobicity to hydrophilicity. This wettability change was reversible, and the hybrid surfaces could return to their original superhydrophobic state upon drying the hydrogels. The authors also created a surface with “reverse response” by embedding the nanowire array into the hydrogels. The setae array could undergo dynamic rearrangement in the dry and wet state. This unique feature resulted in a reversibly variation of the surface wettability from the hydrophilic to the superhydrophobic state. Such a simple design principle may have promising applications in a series of conditions.

### 4.3 Organohydrogel Surfaces with Adaptive Superwettability

Many living creatures have evolved adaptive surfaces by simultaneously employing opposing components (e.g., water and fat molecules) so that they can survive in harsh environments. Inspired by such functionality, Gao et al. developed an organohydrogel through in situ polymerization of oleophilic polymer (butyl methacrylate (BMA) and lauryl methacrylate (LMA)) within a cross‐linked hydrophilic network (*N,N*‐dimethylacrylamide: DMA) swollen with amphiphilic solvents (ethanol) (Figure 5e).^56^ The prepared organohydrogel with a oleophilic/hydrophilic polymer heteronetwork had a high tendency to reconfigure its surface in response to the adjacent liquid phase. In water, the organohydrogel performed like a hydrogel due to the swelling of the hydrophilic network. Conversely, the organohydrogel behaved like an organogel because of the oil absorption by the oleophilic network. Correspondingly, the surface wettability of the organohydrogel could be reversibly switched: it was superoleophobic underwater with an oil CA of ≈156° (hydrogel state) and became superhydrophobic under oil with a water CA of ≈154° (organogel state). More recently, by using hydrophilic monomers (AAm and AA), oleophilic monomers (stearyl methacrylate), as well as oleophilic cross‐linkers (ethylene glycol dimethacrylate), an organohydrogel that can reversibly switch between hydrogel‐ and organogel‐dominated surface reconfigurations has been developed to achieve adaptive superhydrophilic and superoleophilic transitions.^57^ Such adaptive wettability demonstrated excellent reversibility after alternately immersing the organohydrogel in water and oil. This concept of complementary heteronetworks within a gel matrix has inspired researchers to develop soft materials with extraordinary functions.

### 4.4 Superhydrophobic Hydrogel Surfaces

Superhydrophobic hydrogel surfaces have been reported by chemical modification of polymer chains^58^ or the physical introduction of micro/nanoparticles into hydrogel surfaces,^52^, ^59^ while the superhydrophilic networks are well preserved. Yao et al. fabricated the hydrophilic hydrogel via the copolymerization of NIPAAm, clay nanosheets, and dimethylaminopropyl acrylamide, which provided postmodulable active sites.^58^ Then the *n*‐alkylation reaction at the hydrogel/oil interface was performed by immersing the hydrophilic hydrogels into the oil phase containing lodoalkanes or poly(4‐bromomethyl styrene). Such a confined surface modification strategy ensures that the postmodification only occurs on the hydrogel surfaces while maintaining the superhydrophilicity of the intrinsic networks. After modification, the hydrophilic hydrogel surfaces become superhydrophobic. In contrast, the hydrogel surfaces without modification continue to retain their hydrophilicity. Oliveira et al. presented superhydrophobic hydrogels by coating a layer of hydrophobic microparticles on gelatin methacryloyl hydrogel surfaces.^59^ These freestanding hydrophobic hydrogels could float on the surface of aqueous media and be integrated into floating microdevices in many unconventional applications.

### 4.5 Bioinspired Slippery Gel Surfaces

By replacing the water phase in hydrogels with organic phases, organogel surfaces^60^ have been developed to achieve the low adhesion and easy‐sliding properties toward water droplets in air. The criterion for achievement of the easy‐sliding property has been proposed in previous studies, and the following condition should be satisfied^23^

(3) $$\Delta \gamma \;\;=\;\;\gamma_{1}\text{cos}\theta_{4}-\gamma_{2}\text{cos}\theta_{5}-\gamma_{12}>0$$

where γ_1_ and γ_2_ are the surface tension of the infused liquid and the test liquid, respectively. γ_12_ is the interfacial tension between these two liquids. θ_4_ and θ_5_ are the CA of the infused liquid and the test liquid on the solid (with the air around), respectively. It is worth noting that slippery liquid‐infused porous surfaces (SLPSs),^61^, ^62^ which employ similar design principles to gel surfaces, are not discussed herein. The SLPSs are usually overcoated with a thin lubricant layer, which can provide a homogeneous and smooth interface to reduce hysteresis, to repel target immiscible liquids. In contrast to gel surfaces, SLPSs have high a requirement for porous materials to trap infused lubricants and are rarely used for smart surfaces. The readers are referred to recently published reviews for more detailed information.^63^, ^64^, ^65^

**Figure 6:**
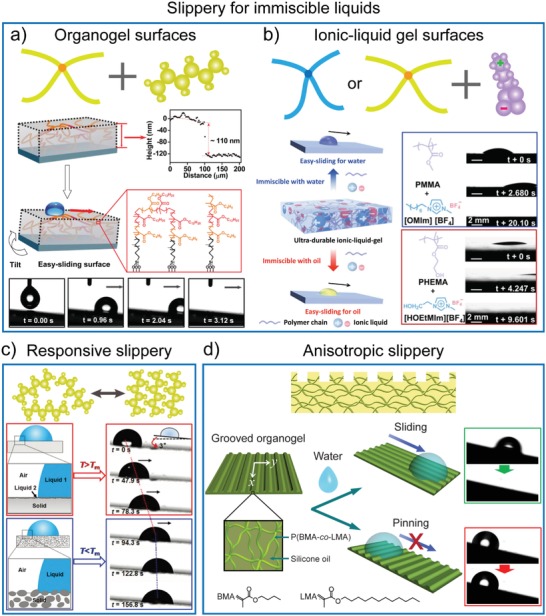
Bioinspired slippery gel surfaces. a) Organogel film with easy‐sliding of water droplets. Reproduced with permission.^23^ Copyright 2013, John Wiley & Sons, Inc. b) Programmable ionic‐liquid‐gels for easy‐sliding of various immiscible liquids. Reproduced with permission.^68^ Copyright 2015, John Wiley & Sons, Inc. c) Thermoresponsive organogel surface. The switching of water between pinning and sliding states on *n*‐paraffin‐swollen organogel can be controlled via the thermoresponsive phase change in *n*‐paraffin. Reproduced with permission.^69^ Copyright 2014, John Wiley & Sons, Inc. d) Grooved organogel surfaces for anisotropic sliding of water droplets. Reproduced with permission.^71^ Copyright 2014, John Wiley & Sons, Inc.

According to Equation (3), Liu et al. introduced, for the first time, the organogel on substrates as a surface coating and achieved the low adhesion to water in the air (**Figure** **6**a).^23^ The cross‐linked polymer networks were synthesized through free radical copolymerization of methacrylate monomers (BMA and LMA) on substrates with an alkene‐terminated surface. The organogel surfaces were obtained by swelling the cross‐linked polymer networks with low viscosity silicone oil (20 cSt). Water droplets immediately slid off the organogel surfaces at a small tilt angle (TA, ≈5°). Simultaneously, contaminants, such as dust, could be removed along with the sliding water. By impregnating the silicone layer‐coated surfaces with silicone oil, Eifert et al. developed a simple and fast method to fabricate organogel surfaces on several materials.^66^ These surfaces showed robust low CA hysteresis (below 4°) even after storing the surfaces for two weeks in a vertical position. By combining magnetic Fe_3_O_4_ nanoparticles armed with dopamine molecules and the copolymer of glycidyl methacrylate and polydimethylsiloxane propyl ether methacrylate, Jin et al. prepared a silicone oil swollen organogel by reversible coordinate bonds and strong covalent bonds. The organogel surfaces could remarkably recover after damage, due to the synergistic interactions of reversible Fe‐catechol coordination and diffused lubricating liquid.^67^

To repel various liquids, not just water, Ding et al. developed ultradurable ionic‐liquid‐gel (ILG) surfaces for easy‐sliding of both water and organic droplets (Figure 6b).^68^ For example, ILG fabricated using poly(methyl methacrylate) and 1‐octyl‐3‐methylimidazolium tetrafluoroborate ([OMIm][BF_4_]) achieved easy‐sliding of immiscible water at a TA of 10°. ILG composed of PHEMA and 1‐hydroxyethyl‐3‐methylimidazolium tetrafluoroborate ([HOEtMIm][BF_4_]) demonstrated an easy‐sliding property for immiscible decane droplets. Importantly, the surface properties of ILG can be adjusted according to the target liquids by manipulating the composition of the polymer chains and ILs. Owing to the negligible volatility of ILs as well as the stable polymer networks, the easy‐sliding property can be maintained after exposure in air for over eight months, and it can endure temperatures as high as 280 °C. Such ultradurable ILG surfaces would greatly promote practical applications of easy‐sliding surfaces.

The transition of the solvents inside the gels between different states has also been utilized to design responsive organogel‐based slippery surfaces. For example, *n*‐paraffin can undergo a change from the solid phase to the liquid phase when increasing the temperature from below its melting temperature (*T*_m_) to above its *T*_m_. By using such a thermos‐responsive phase change of *n*‐paraffin, Yao et al. designed the *n*‐paraffin‐swollen polydimethylsiloxane (PDMS) organogel, on which the movement of water droplets could be thermally switched (Figure 6c).^69^ At a temperature below *T*_m_ (25 °C), the organogel was milky‐white due to the solid‐state of *n*‐paraffin. Water droplets were pinned on such surfaces. By increasing the temperature to 75 °C (above *T*_m_), a liquid layer could be formed on the organogel surface owing to the melted liquid alkane. In this case, water droplets could easily slide off at a small TA (≈6°). Such switchable slippery performance demonstrated excellent reversibility during heating/cooling cycles. Additionally, the switching temperature could be tuned by selecting alkanes with different *T*_m_ for the desired range as the requirement for practical applications. A similar organogel surface was fabricated by impregnating a nanostructured surface with a gelator (12‐hydroxystearic acid (12‐HAS)) containing lubricant (mineral oil).^70^ Mineral oil gelled with 12‐HAS was intrinsically thermos‐responsive as the viscosity of the organogel varied with temperature. The resulting organogel surface exhibited a thermally controllable water sliding property.

By introducing anisotropic structures into organogel surfaces, we achieved excellent anisotropic slippery surfaces for water droplets in a very simple manner (Figure 6d).^71^ The organogels with anisotropic periodic microgrooves were fabricated on silicon templates with periodic microgrooves using one‐step radical copolymerization of BMA, LMA, and silicone oil. Water droplets could easily slide along the direction parallel to the microgrooves due to the formed slippery water/oil interface, but they pinned on the surface when perpendicular to the micro‐grooves at a TA of ≈10°. Furthermore, owing to the elastic property of organogels, unidirectional sliding of water droplets was also achieved by asymmetrically stretching the microgrooved organogel surfaces. In contrast to the existing anisotropic superhydrophobic surfaces^72^, ^73^ and gradient surfaces,^74^, ^75^, ^76^, ^77^ the grooved organogel surfaces provide a versatile candidate for the anisotropic sliding of water droplets by introducing a slippery water/oil interface and may significantly promote the broad application of anisotropic sliding of liquids in various fields.

### 4.6 Superspreading of Miscible Liquids on Immersed Gel Surfaces

**Figure 7:**
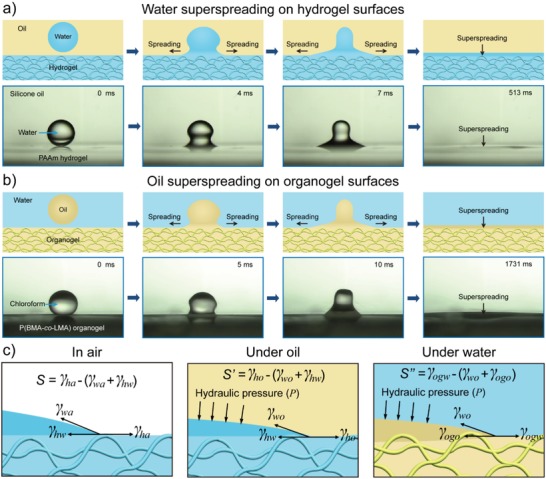
Superspreading of miscible liquids on immersed gel surfaces. Illustration and time‐lapse images of the superspreading process of a) a water droplet on hydrogel surfaces in an oil/water/hydrogel system and b) a chloroform droplet on the chloroform‐swollen P(BMA‐*co*‐LMA) organogel surface. c) Illustrations of the spreading coefficient *S* of liquid droplets spreading on gels in air, under oil, and underwater. Reproduced with permission.^78^ Copyright 2016, John Wiley & Sons, Inc.

When investigating the wetting behaviors of miscible liquids on immersed gel surfaces, we observed the superspreading phenomenon in the liquid/liquid/gel system (**Figure** **7**).^78^ Previous literature has demonstrated that water droplets are unable to completely spread on hydrogel surfaces in air.^79^, ^80^ Indeed, we observed a similar phenomenon on Ca‐alginate, PAA, poly(vinyl alcohol) (PVA), and PAAm hydrogel surfaces in air. However, when immersing the PAAm hydrogel in silicone oil, we observed superspreading of water droplets on the PAAm hydrogel surfaces (Figure 7a). Similarly, superspreading of a series of miscible oils and liquid monomers and even high viscosity liquids was achieved on the immersed P(BMA‐*co*‐LMA) organogel surfaces (Figure 7b). The achievement of such superspreading behaviors was reliant on the liquid‐like property of gel surfaces and the miscibility between spreading liquids and the liquid phases inside the gels and the introduction of the liquid phase, which significantly increased the spreading coefficient and provides hydraulic pressure (Figure 7c).

## 5 Emerging Applications

The superwettability and special adhesion of bioinspired gel surfaces endows them with practical promise in various fields, such as liquid/liquid separation, antiadhesion of organisms and solids, and fabrication of functional thin polymer films. This section will introduce these emerging applications in detail.

### 5.1 Liquid/Liquid Separation

**Figure 8:**
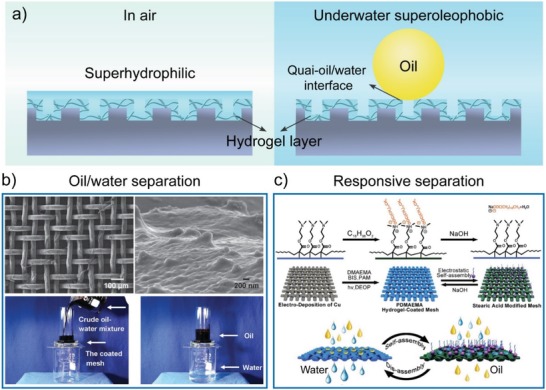
Bioinspired hydrogel surfaces with superwettability for liquid/liquid separation. a) Structured hydrogel surfaces are superhydrophilic and underwater superoleophobic. b) The PAAm hydrogel‐coated stainless steel mesh with underwater superoleophobicity and low oil‐adhesion for efficient separation of oil/water mixtures. Water selectively permeates through the mesh, while the oil is repelled and kept in the upper glass tube. Reproduced with permission.^22^ Copyright 2011, John Wiley & Sons, Inc. c) Hydrogel surfaces for pH‐responsive oil/water separation. The PDMAEMA hydrogel‐coated mesh can switch between superhydrophilicity/underwater superoleophobicity and superhydrophobicity/‐superoleophilicity through self‐assembly and disassembly of stearic acid in solutions without or with NaOH. Reproduced with permission.^47^ Copyright 2014, Royal Society of Chemistry.

The development of novel materials for efficient oil/water separation is an urgent need for environmental and economic demands because of the frequent oil spill accidents and the rapid increase in oily industrial wastewater.^81^ The materials' surfaces with superwettability and special adhesion toward water and various oils have attracted considerable attention and may provide an efficient and facile way to address the above problems.^11^, ^14^, ^82^ Hydrogels have become promising candidates and exhibit outstanding capacity for efficient oil/water separation due to their superhydrophilicity in air and superoleophobicity and low oil‐adhesion underwater (**Figure** **8**a). By utilizing such unique properties, Xue et al. developed a PAAm hydrogel‐coated stainless steel mesh, which could selectively and efficiently remove water from oil/water mixtures, such as gasoline, diesel, vegetable oil, and even crude oil/water mixtures (Figure 8b).^22^ These meshes allowed water to permeate through the mesh driven by gravity but obstructed oil by a water barrier formed at the hydrogel surface. Due to the underwater superoleophobicity and low oil‐adhesion property, the hydrogel‐coated meshes also possessed resistance to oil fouling. This innovative study provided a simple way for the design of next‐generation functional materials for efficient oil/water separation. Since then, a series of hydrogels have been fabricated and used for separation of the oil/water mixture, significantly promoting the development of advanced separation techniques.^83^, ^84^, ^85^, ^86^, ^87^, ^88^, ^89^, ^90^ For instance, Yan et al. developed a facile yet effective way to fabricate hydrogel‐coated meshes for efficient separation of a wide range of oil‐in‐water mixtures.^90^ The Fe/polylactic acid‐based meshes were fabricated by using fused deposition modeling 3D printing. The hydrogel coatings were prepared through in situ polymerization of the Fe(II)‐mediated redox reaction and infused with aluminum chloride, which was attributed to strengthening of the demulsification of oil‐in‐water emulsions, leading to efficient separation of the oil‐in‐water mixtures.

Practically, the composition of oil/water mixtures is exceptionally complicated, such as seawater with high salt, oily industrial wastewater with a high temperature, high acid, or high alkalinity, and surfactant‐stabilized emulsions. These harsh conditions cause the oil/water separation to encounter severe challenges. Several hydrogels have been used to address these challenges.^91^, ^92^, ^93^, ^94^, ^95^, ^96^, ^97^ For example, Chen et al. developed a silica gel‐coated quartz fiber mesh for oil/water separation under strong acidic and concentrated salt conditions.^91^ A poly(3, 4‐ethylene dioxythiophene)–poly(styrene sulfonate) hydrogel‐coated titanium mesh was fabricated to selectively and effectively separate a series of mixtures of oil and various corrosive aqueous solutions, including strong acidity, alkaline, high salt, and hot‐water with up to 99.9% separation efficiency.^92^ Notably, Fan et al. developed a simple method to prepare hydrogel‐coated filter papers for separating the oil/water mixture with high acidity, high alkalinity, high salinity, and surfactant‐stabilized emulsion.^93^ The hydrogel‐coated cellulose filter paper was fabricated by direct cross‐linking of PVA to cellulose using glutaraldehyde as the cross‐linker. Benefiting from the formed multiple cross‐linked networks, the as‐prepared hydrogel‐coated filter paper was robust for the separation of oil/water mixtures containing high acidity (8 m H_2_SO_4_), alkalinity (10 m NaOH), or saturated NaCl with >99% separation efficiency. Recently, a poly(2‐acrylamido‐2‐methyl‐1‐propanesulfonic acid) (PAMPS) hydrogel‐coated cellulose membrane was used for highly efficient and selective separation of cationic and nonionic types of surfactant‐stabilized emulsions via hydrogen bond interactions between surfactants and the sulfonic groups in the polymer chains of the PAMPS hydrogel.^97^ Such hydrogel‐coated membranes can be easily scaled up and facilitate their practical applications in various fields, including sewage remediation.

Stimuli‐responsive hydrogels have been used to achieve intelligent and controllable liquid/liquid separation. pH, an essential feature for aqueous solutions, was employed to control the separation of oil/water mixtures.^47^, ^98^ Through pH‐controlled self‐assembly and disassembly of stearic acid, the PDMAEMA hydrogel‐coated stainless steel mesh could selectively remove water or oil from oil/water mixtures with high efficiency (Figure 8c).^47^ Due to the thermoresponsiveness of PDMAEMA, the PDMAEMA hydrogel‐coated mesh could allow water and oil to permeate through the mesh sequentially by adjusting the temperature or pH. Under 55 °C (pH 7) and at a pH lower than 13 (25 °C), the PDMAEMA hydrogel‐coated mesh only permitted the passage of water, thus removing water from oil/water mixtures. Increasing the temperature above 55 °C or the pH greater than 13 allowed the oil to permeate and be collected in situ. Thermoresponsive oil/water separation was also achieved by PNIPAAm hydrogel‐coated polyurethane (TPU) microfiber webs.^99^ The TPU‐PNIPAAm membrane could switch from superhydrophilicity to superhydrophobicity along with the increase in temperature from 25 to 45 °C, leading to the highly efficient separation of 1 wt% oil‐in‐water emulsion and 1 wt% water‐in‐oil emulsion at 25 and 45 °C, respectively. A novel Hg^2+^‐responsive PAA hydrogel‐coated mesh was reported for the purification of oil‐polluted water containing the Hg^2+^ contaminant.^100^ When Hg^2+^ was present in the oil/water mixture, the PAA hydrogel‐coated mesh became hydrophobic and oleophilic due to the chelation between mercury and PAA, leading to the penetration of oil through the mesh, but the obstruction of water. These stimuli‐responsive hydrogel surfaces shed light on the development of intelligent devices for sequential liquid/liquid separation.

### 5.2 Antibiofouling

**Figure 9:**
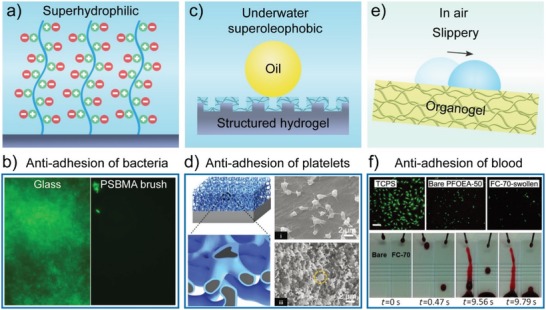
Bioinspired gel surfaces with superwettability for antibiofouling. a) Illustration of the superhydrophilicity of zwitterionic polymer brushes through ionic solvation. b) Zwitterionic PSBMA brushes for effectively prohibiting bacterial adhesion. Reproduced with permission.^109^ Copyright 2007, Elsevier Ltd. c) Illustration of the underwater superoleophobicity of structured hydrogel surfaces. d) Porous NiTi/hydrogel surfaces for antiplatelet adhesion. Reproduced with permission.^117^ Copyright 2017, John Wiley & Sons, Inc. e) Illustration of slippery organogel surfaces for easy‐sliding of water droplets. f) Organogel surfaces exhibit outstanding antiadhesion of cells and blood. Reproduced with permission.^118^ Copyright 2014, John Wiley & Sons, Inc.

Organisms, such as proteins, cells, bacteria, and mussels, have become a severe issue for many types of equipment used in aqueous environments, including implants, biomedical devices, and marine ships. Bioinspired gel surfaces have been intensively used as antibiofouling coatings. Although the antibiofouling property of hydrogels has been used for a long time, they still fail to meet the requirements of practical applications, such as long‐term performance and durability in harsh environments.^101^, ^102^, ^103^, ^104^, ^105^, ^106^ Several chemical and physical strategies have been employed to improve their performance. Jiang's group proposed a novel antibiofouling avenue by endowing the surfaces with a homogenous mixture of balanced charge groups from zwitterionic or mixed charged polymers, which could form a strong surface hydration layer through ionic solvation (**Figure** **9**a).^107^, ^108^ They have developed a series of antibiofouling surfaces by using either zwitterionic polymer brushes^102^, ^109^ or hydrogels,^110^, ^111^, ^112^, ^113^ including poly(carboxybetaine), poly(carboxybetaine methacrylate), poly(sulfobetaine) (PSB), and poly(sulfobetaine methacrylate) (PSBMA). Such superhydrophilic zwitterionic surfaces exhibited outstanding antiadhesion performance toward various proteins, bacteria, cells, and marine fouling (Figure 9b).

Surface hydration can also be enhanced to create robust liquid‐like surfaces by coating hydrogel layers onto solid surfaces with a micro/nanoscale topography. The resulting structured gel surfaces exhibiting underwater superoleophobicity have been widely used to design advanced antibiofouling surfaces (Figure 9c). For instance, Chen et al. reported a nanostructured surface by grafting PNIPAAm brushes on silicon nanowire arrays (SiNWA).^114^ The SiNWA–PNIPAAm surfaces exhibit significantly reduced adhesion of platelets, compared with smooth PNIPAAm surfaces. The SiNWA–PNIPAAm surfaces show underwater superoleophobicity and low oil‐adhesion, indicating that the nanostructured surfaces can retain a relatively high ratio of water content, which is responsible for the primarily reduced adhesion of platelets. Zhang et al. fabricated fibrillar anionic and zwitterionic hydrogel brushes using anodic aluminum oxide (AAO) as a template to improve the antibiofouling property.^115^ They introduced anionic 3‐sulfopropyl methacrylate (SPMA) and zwitterionic SBMA polymers into the PAA hydrogel network to synthesize fibrillar hydrogel surfaces (P(AA/SPMA) and P(AA/SBMA)) with a length of 3–4 µm and diameter of ≈100 nm. These fibrillar hydrogel surfaces exhibited underwater superoleophobicity and extremely low oil adhesion. Notably, compared with AAO, such surfaces could effectively prohibit adhesion of algae: 90% and 95% decrease in fouling algae (*Dunaliella tertiolecta* and *Navicula* sp.) for P(AA/SPMA) and P(AA/SBMA) hydrogel surfaces, respectively. The formed stable hydration layers of the structural hydrogels contribute to the outstanding antibiofouling performance.^102^ Through the self‐assembly of hierarchical microgel spheres, Chen et al. presented a self‐repairing underwater superoleophobic and antibiofouling coating.^116^ The combination of ordered surface structures and hydrogels caused the prepared surfaces to exhibit outstanding underwater oil‐repellant and biofouling‐resistant properties (BSA adsorption of ≈3 µg cm^−2^). Importantly, this hydrogel‐based coating displayed excellent underwater self‐healing capability once mechanically damaged, indicating its potential applications for long‐term antibiofouling in marine or other aqueous environments. Hydrogels usually must be bonded to hard substrates such as metals and ceramics to improve mechanical durability. Using this strategy, Zang et al. fabricated the porous NiTi/hydrogels nanocomposites by photopolymerizing the PAAm and PVA hydrogel with an ≈100 nm thickness on TiO_2_ nanolayer‐coated porous NiTi (Figure 9d).^117^ In comparison to pristine NiTi, porous NiTi/hydrogel nanocomposites have the same level of mechanical durability and integrity after 100 cycles of bending and twisting tests. Additionally, the high‐water content and hierarchical structures of such nanocomposites contributed to their superoleophobicity, ultralow oil‐adhesion, and exceptional antiplatelet properties, leading to their advanced technological promise in endovascular devices such as vena cava filters and coronary stents.

In addition to hydrogel surfaces, slippery organogel surfaces are promising candidates for the development of antibiofouling surfaces due to the significantly reduced adhesion between organisms and liquid‐like surfaces (Figure 9e). Yao et al. fabricated lubricant‐swollen organogels using photocuring perfluorinated alkyl acrylate monomers (2‐perfluorooctylethyl acrylate or 2‐perfluorohexylethyl acrylate) and a fluorinated macromolecular cross‐linker (perfluoropolyether dimethacrylate) and subsequent swelling with lubricants (FC‐70) (Figure 9f).^118^ The organogel surfaces were homogeneously wetted and had large amounts of free polymer chain ends, facilitating the reduction of interfacial adhesion and friction. Compared with tissue culture polystyrene, organogel surfaces could significantly inhibit the adhesion and spreading of mouse embryonic fibroblast cells and significantly reduce the adhesion of blood, supporting their great potential as antibiofouling surfaces for biomedical devices. Similarly, by infusing silicone oil into PDMS with vascular networks, Howell et al. reported an organogel‐based biofouling‐release surface.^119^ The resulted organogel surface could significantly inhibit the colony formation of *S. aureus*, microalgae *B. braunii*, *C. reinhardtii*, *D. salina*, *N. oculata*, and *E. coli* for long‐term testing (2–14 days). The silicone oil infused PDMS organogel surfaces demonstrated ultralow preferential attachment and ultralow adhesive strengths for mussels in marine fields over 16 weeks.^120^ The authors claimed that the organogel surface could deceive the mechanosensing ability of mussels, prohibit the secretion of sticky threads, and reduce the molecular work of adhesion. Such organogel surfaces represent a promising candidate to address marine biofouling‐induced economic and ecological issues.

### 5.3 Antisolid Adhesion

**Figure 10:**
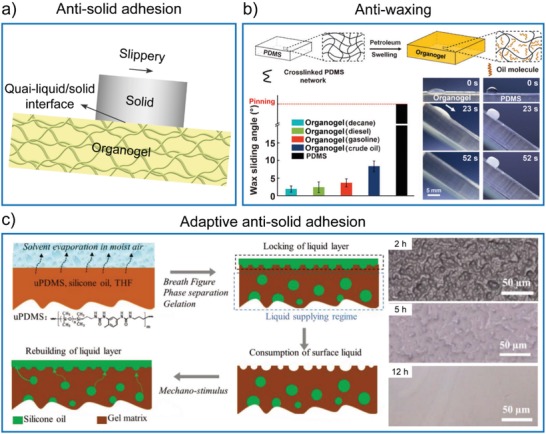
Bioinspired slippery organogel surfaces for antisolid adhesion. a) Illustration of slippery organogel surfaces for antiadhesion of solids through the formation of a quasi‐liquid/solid interface. b) PDMS organogel surfaces for antiadhesion of solidified paraffin wax. Reproduced with permission.^127^ Copyright 2015, John Wiley & Sons, Inc. c) Self‐replenishing organogel surface for adaptive antisolid adhesion by the stimulus‐responsive release of liquids. Reproduced with permission.^135^ Copyright 2018, John Wiley & Sons, Inc.

Excluding liquids and organisms, matters in solid‐states, such as ice, snow, paint, and wax, interact with gel surfaces at high frequency in practical applications and may cause severe problems in aviation, space flight, radar, transport, and oil pipelines. Due to their liquid‐like property, organogel surfaces have exhibited outstanding performance for antiadhesion of various solids (**Figure** **10**a).^121^ Of note, the hydrogels used for adhesives were reviewed recently^122^, ^123^, ^124^, ^125^ and will be not discussed herein. For instance, organogel surfaces have been used for antiwaxing, which has technical promise to address the issue of wax deposition in industrial petroleum pipelines.^126^ Yao et al. presented a self‐replenishable PDMS organogel swollen with petroleum, showing sustainable ultralow adhesion to solidified paraffin wax and crude oil by absorption of low‐molar‐mass oil from its crude‐oil environment (Figure 10b).^127^ Due to the existence of the thin oil surface layer, the adhesion of solidified paraffin wax on the organogel surface was more than 500 times lower than that of conventional material surfaces. The shear stress of the wax was only 0.5 kPa on such organogel surface, leading to its easy‐sliding under flow conditions.

PDMS organogels infused with various liquids were also used for anti‐icing. Zhu et al. fabricated a silicone oil‐infused PDMS organogel, on which the shear adhesion strength of the ice was ≈40 kPa compared with 55 kPa of PDMS without silicone oil.^128^ Wang et al. reported a liquid‐paraffin swollen PDMS organogel presenting an ultralow ice‐adhesion strength of 1.7 ± 1.2 kPa (at −30 °C).^129^ A lower ice adhesion strength of self‐lubricating organogel was also prepared. By tuning the compatibility of the impregnated organic liquids with the PDMS network, the syneresis of liquids from the organogel matrices endowed the organogel surface with excellent and sustainable anti‐icing functions, with an ice adhesion strength reaching as low as 0.4 kPa.^130^ This surface also had antisticking properties against several viscous emulsions (mayonnaise, honey, ketchup, liquid glue, and Worcester sauce) and their dry solidifications. Recently, through tailoring the cross‐link density of PDMS and embedding miscible polymer chains to enable interfacial slippage, Golovin et al. obtained a durable anti‐icing organogel with extremely low ice adhesion of 0.15 kPa.^131^ The prepared organogels could act as extremely durable coatings that maintain low ice adhesion (<10 kPa) in harsh conditions such as severe mechanical abrasion, acid/base exposure, 100 icing/deicing cycles, and exposure to wintery conditions over several months.

An organogel surface with adaptive antisolid adhesion property was also developed by mimicking earthworms, which could pass through adhesive soil without strains due to the unique self‐lubricating layer on their rough skin. This feature is attributed to the epidermal glands under the skin of earthworms. Under external mechanical stimuli, the epidermal glands can continually secrete mucus, which can be stabilized by the rough bark, leading to the formation of a thick and slippery layer.^132^, ^133^ This extraordinary ability inspired scientists to prepare artificial surfaces with similar functions.^134^, ^135^ Recently, Cui and co‐workers reported a self‐replenishing organogel coating, made from the silicone oil swollen copolymer of urea and PDMS (uPDMS) (Figure 10c).^135^ The uPDMS organogel had textured surfaces to entrap liquids and droplet‐embedded bulk structures that permitted self‐regulated liquid release. Similar to the earthworms, the organogel coating had an adaptive “secretion” capability. It could release the lubricant stored inside the droplets under an external mechanical stimulus to restore the oil layer in a rapid and site‐specific manner, thus maintaining reduced friction of the surfaces in a solid‐based friction environment. By swelling PDMS with alkane tetracosane, which has a melting temperature higher than room temperature, a novel organogel with the regenerable sacrificial surface was developed.^136^ Unlike the liquid layer, the solid surface layer neither evaporated nor contaminated nearby surfaces. The solid surface layer was regenerable: alkane stored inside the polymer matrix could diffuse out to form a new surface layer after the initial one was damaged. The organogel with a solid surface layer displayed excellent antiadhesion for not only complex liquid contaminants such as honey, jam, and blood but also solid depositions such as graffiti, paints, and ice. The ice adhesion strength on the solid organogel surface was remarkably reduced to 68.8 ± 10.4 kPa and remained unchanged after 20 icing/deicing cycles.

### 5.4 Antifriction

**Figure 11:**
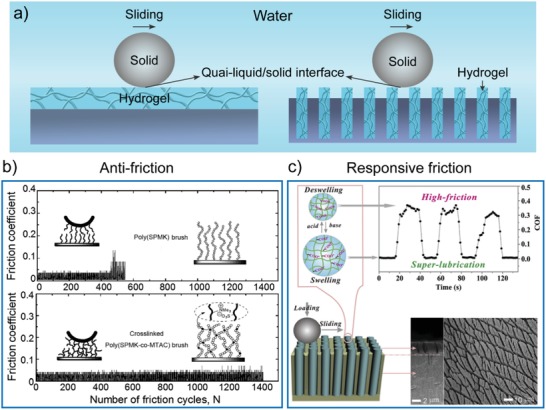
Bioinspired composite hydrogel surfaces for antifriction. a) Illustration of slippery hydrogel surfaces for antifriction through the formation of a quasi‐liquid/solid interface. b) Evolution of the friction coefficient versus the number of friction cycles for surfaces with polymer brushes (top) and cross‐linked hydrogels (bottom). Reproduced with permission.^153^ Copyright 2012, Royal Society of Chemistry. c) Nanoporous substrate‐infiltrated hydrogels for high load bearing and tunable friction. Reproduced with permission.^164^ Copyright 2016, Elsevier Ltd.

Articular cartilage has a hydrogel‐like surface with a low‐friction‐coefficient (0.001–0.03), supported by aligned collagen fibers.^137^, ^138^ Great efforts have been made in terms of the design and production of artificial surfaces with low friction property.^38^, ^139^, ^140^, ^141^, ^142^, ^143^, ^144^, ^145^, ^146^ Polymer brushes have been primarily tethered onto solid surfaces to reduce interfacial friction. Klein and co‐workers reported a series of polymer brushes and developed a molecular model to reveal the underlying mechanism.^38^, ^142^, ^143^, ^147^, ^148^, ^149^, ^150^ The concept of hydration lubrication they proposed has been invoked to account for the extremely low sliding friction observed at high pressures between charged surfaces in high‐salt solution.^36^, ^37^, ^151^ Water in the hydration layer is tenaciously held and may provide very rapid relaxation. Thus, it is difficult to squeeze the hydration water out under compression between two surfaces. In addition, the hydration shells respond in a fluid‐like manner, and the surfaces may slide easily past each other when sheared. This combination of supporting a large compressive load together with easy‐sliding enables the hydration layers to act as excellent lubrication elements in aqueous surroundings. However, one limitation of polymer brushes is their low wear resistance. Thus, cross‐linked polymer brushes were developed to address the above challenge (**Figure** **11**a).^152^, ^153^, ^154^, ^155^, ^156^ Li et al. fabricated a PAAm thin film with cross‐linked polymer networks to improve the wear resistance.^152^ By comparison, the PAAm polymer brushes were also prepared. Compared with the PAAm brushes, which behaved as highly effective lubricating films when immersed in an aqueous environment, the hydrogel brushes showed an increased coefficient of friction due to progressive elimination of the lubricious brush structure coupled with reduction of the water content of the films. However, the hydrogel brushes had a higher Young's moduli, indicating the improved wear performance. By surface‐initiated controlled radical copolymerizations of 2‐(methacryloyloxy)ethyltrimethylammonium chloride) (MTAC) and 3‐sulfopropyl methacrylate potassium salt (SPMK), Kobayashi et al. reported cross‐linked poly(SPMK‐*co*‐MTAC) hydrogel brushes, showing stable low friction (Figure 11b).^153^ Compared with noncross‐linked poly(SPMK) brushes, cross‐linked poly(SPMK‐*co*‐MTAC) hydrogel brushes continuously showed an extremely stable friction coefficient of ≈0.015 in water after 1400 friction cycles even under a regular load of 139 MPa. The cross‐linked structures improved the shear strength and wear resistance of the polymer brush, leading to a long period of more than 1000 friction cycles even under severe pressure. Recently, Iuster et al. presented cross‐linked poly[2‐(methacryloyloxy)ethylphosphorylcholine] hydrogel layers exhibiting very low friction (friction coefficients of 10^−3^–10^−4^), similar to the corresponding linear brushes.^156^ Importantly, they found a marked difference between the noncross‐linked brushes and cross‐linked hydrogel layers in the dependence of friction on the sliding velocity versus For the noncross‐linked brushes, the shear force remained constant mainly along with the increase in typical loads. By contrast, the shear force increased with an increasing shear rate for the cross‐linked brushes, an effect attributed to the suppression of interpenetration by cross‐linking. Recently, Ma et al. developed a versatile method for the rapid fabrication of functional hydrogel coatings on the surfaces of various complex structures by catalytically initiated radical polymerization on surfaces, which occurred through the redox reaction between Fe^2+^ and S_2_O_8_^2−^ at the solid/liquid interface.^157^ This method enabled good control over the chemical components, thickness, and network structures of the hydrogel layers, easily changing the surface wettability, and lubrication properties. Importantly, this method is also compatible with existing 3D‐printing technology for engineering hydrogel‐coated hollow complex structures.

Although hydrogels have a wide range of friction coefficients from 10^−4^ to 10^°^,^158^ conventional hydrogels are too weak to be practically used in high bearing conditions. To solve this problem, Gong's group reported mechanically robust hydrogels by using a double‐network structure.^159^ The double‐network hydrogel had an ultralow friction surface (friction coefficients as low as 10^−5^) and a fracture strength as high as 9 MPa. A series of composite hydrogels were also developed by Zhou's group to mimic the design principle of articular cartilage.^160^, ^161^, ^162^, ^163^, ^164^, ^165^ For example, a robust bilayer hydrogel was prepared by interfacial modulated polymerization.^162^ The top layer with a low‐density cross‐linked network ensured ultralow friction, while the bottom matrix with a high‐density cross‐linked network rendered a high load‐bearing property. This bilayer structure of the hydrogel achieved low and durable friction (friction coefficient of 0.01–0.05) under a high bearing load of 5–30 N. Zhang et al. synthesized a hydrogel by using a thixotropic supramolecular network and double network structure, showing unique shear‐responsive lubricating properties.^166^ The shear force induced disassembly of the *N*‐fluorenylmethoxycarbonyl‐l‐tryptophan supramolecular network endowed the hydrogel with a lubricating function (friction coefficient of 0.02). Moreover, the PAAm/PVA double network acted as the supporting framework with high mechanical strength.

By using stimuli‐responsive hydrogels, the lubrication of solids can be controlled on‐demand. For instance, by integrating ordered PAA hydrogel nanofibers into AAO templates, heterogeneous structures were prepared (Figure 11c).^161^ In such a design, the soft hydrogel fibers provided excellent aqueous lubrication, and the hard AAO offered a high load‐bearing capacity, resulting in a low friction coefficient (<0.01) under high contact pressures at the level of MPa. Moreover, the friction could be switched between high (>0.3) and superlubrication (≈10^−3^) by using stimuli‐responsive hydrogels.^160^, ^167^ The composite interface showed high friction or low friction in acid or basic media, respectively. The friction switching occurred very fast by alternatively wetting with weak acid media (pH 3, below the pKa of PAA) and weak basic media (pH 10, above the pKa of PAA). The graphene oxide/PNIPAAm composite hydrogels had switchable friction in response to external temperature stimuli.^167^ Below the LCST, the composite hydrogel in the swelling state showed an ultralow friction coefficient (≈0.03). By contrast, the composite hydrogel had a high friction coefficient (≈0.49) in the shrinking state above the LCST. By incorporating sodium methacrylate or DMAEMA into PNIPAAm hydrogels, the as‐prepared hybrid hydrogels displayed an ultralow friction coefficient (≈0.05).^160^ The stepwise switch of the friction coefficient between 0.05 and 1.2 was achieved through sequential regulation of the pH and temperature. More recently, Ma et al. reported a unique construct consisting of coated double‐side PAA hydrogel nanofibers, the friction properties of which could be reversibly switched simultaneously and separately.^165^ Relying on their swelling and shrinking under basic/acid conditions, these hydrogel nanofibers showed a highly tunable response and three different friction states (friction coefficient ranging from 0.3–0.4 to <0.01). Altogether, these results provide a promising route for the design of superlubrication materials using hydrogels with smart surface/interface properties.

### 5.5 Fabrication of Functional Thin Polymer Films

**Figure 12:**
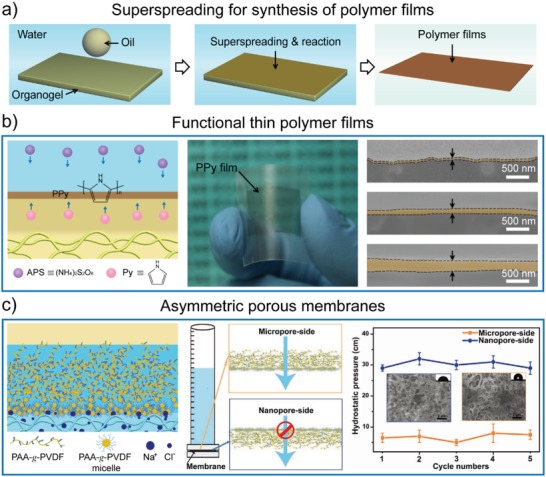
Superspreading of miscible liquids on immersed gel surfaces for the synthesis of functional thin polymer films. a) Illustration of the superspreading‐based fabrication process of thin polymer films. b) Thin PPy films with various thicknesses were fabricated on an immersed organogel surface. Reproduced with permission.^78^ Copyright 2016, John Wiley & Sons, Inc. c) Asymmetric PAA‐*g*‐PVDF membranes synthesized using an interfacial confined phase inversion process on an immersed PAAm hydrogel surface. The prepared PAA‐*g*‐PVDF membranes showed symmetric porous structures and wettability, leading to the directional water flow gating property. Reproduced with permission.^168^ Copyright 2016, John Wiley & Sons, Inc.

The superspreading of liquid droplets on gel surfaces induced the formation of confined, stable, and homogeneous liquid layers, which motivated us to perform an interfacial reaction and fabrication by introducing the reactants into the liquid phase, superspreading liquid layers, and/or gels (**Figure** **12**a).^78^, ^168^, ^169^ Several kinds of thin polymer films with thicknesses that could be controlled from nanometers to micrometers, were fabricated through one‐step polymerization reactions on the immersed organogel surfaces. For example, conducting thin polypyrrole (PPy) films with various thickness were synthesized on immersed organogel surfaces by separately introducing the reactants into the water phase and spreading oil solutions (Figure 12b).^78^ Compared with traditional interfacial polymerization, the superspreading‐based method could significantly improve the stability of the oil/water interfaces, leading to the uniform surfaces of the prepared PPy films. This strategy has also been utilized to fabricate other functional polymer films, including thin polyamide films, freestanding poly(urethane acrylate) films, and PDMA/nanoclay composite hydrogel films.

The superspreading of aqueous solutions on hydrogel surfaces under oil has also been used to fabricate functional thin films. For instance, asymmetric membranes were synthesized for efficient fluid gating. The asymmetric PAA‐*g*‐PVDF membranes were fabricated by employing the superspreading of the PAA‐*g*‐PVDF solution on PAAm hydrogel surfaces under silicone oil and a salt‐induced phase inversion process (Figure 12c).^168^ This process induced the formation of asymmetric porous structures on the two sides of the as‐prepared PAA‐*g*‐PVDF membranes. The side with microscaled pores was more hydrophobic than the side with nanoscaled pores, leading to the directional water flow gating property. Water could flow from the micropore side to nanopore side at a lower hydrostatic pressure. From the reverse direction, a higher hydrostatic pressure was needed. More recently, Hao et al. reported the confined synthesis of 2D covalent organic framework (COF) thin films by using the superspreading strategy.^169^ They used the superspreading water layer on an immersed hydrogel surface as a reactor and introduced amine monomers (4,4′,4″‐(1,3,5‐triazine‐2,4,6‐triyl)trianiline) and aldehyde monomers (2,5‐dihydroxyterethaldehyde) into the PAAm hydrogel and oil phase (tridecane), respectively. Thin films of COFs with tunable thickness, a homogeneous morphology, crystallinity, and a large area were successfully fabricated after the diffusion of these reactants into the thin superspreading water layers. In addition, this superspreading strategy was extended to the synthesis of other COFs and crystalline zeolitic imidazolate framework‐8 (ZIF‐8) thin film. These studies highlight the promise of the superspreading approach for the development of functional thin polymer films for various applications.

## 6 Conclusion and Perspective

In this review, we summarize recent progress and emerging applications related to bioinspired gel surfaces with superwettability and special adhesion. Biological hydrogel‐based surfaces, including carp scales, filefish skins, seaweed surfaces, the toe of the tree fog, *Nepenthes* pitcher, and articular cartilage, show superwettability and special adhesion, as well as outstanding performance in their native environments. It has been suggested that the achievement of these unique properties is attributed to the unique liquid‐like gel surfaces, which can be engineered by designing polymers, solvents, and surface structures. By using such a design principle, artificial gel surfaces with superwettability and special adhesion have been developed and exhibit unprecedented performance in diverse applications, such as liquid/liquid separation, antibiofouling, antisolid adhesion, antifriction, and the synthesis of functional thin polymer films. Among these, we suggest a new principle for the design and development of next‐generation advanced materials with superior surfaces for practical applications.

Although considerable progress has been made in this field, the investigation of bioinspired gel surfaces with superwettability and special adhesion is still in its infancy, and numerous challenges in both fundamental research and practical applications must be addressed with great efforts. First, the exploration of novel natural livings with functional surfaces, especially those spending most of their lifetime in wet, rainy, or aqueous habitats, is pivotal to enable the discovery of unique mechanisms and design principles for advanced gel surfaces. To achieve this goal, the issues encountered for current gel surfaces, such as the biocompatibility of gels when used in medical devices,^64^, ^65^ have solutions with much higher possibilities.

Second, only a limited number of polymers, solvents, and surface structures have been used to fabricate gel surfaces. There are abundant polymers (supramolecular polymers, conducting polymers, biomacromolecules, etc.), solvents (metallic liquids, magnetic liquids, liquid crystals, etc.), and surface structures (gradient structures, asymmetric structures, multiscale structures, etc.), which could be utilized to design and develop new gel surfaces. Therefore, there are many opportunities to promote the development of advanced gel surfaces by engineering the polymers, solvents, and/or surface structures. Additionally, intensive efforts should be exerted toward the exploitation of novel materials and advanced technologies for the fabrication of bioinspired gel surfaces in a facile, efficient, cost‐effective, and scalable way. Synthetic gel surfaces with multiple functions, such as biocompatibility, environmentally friendly, and long‐term stability/durability under harsh conditions, remain a challenge.

The combination of solid surfaces with water, air, and oil can create a total of 64 wetting states.^1^ However, as shown in Figure 2, only several wetting states toward water and oil have been investigated for gel surfaces to date. Therefore, the superwettability of gel surfaces is far from well understood. We should attribute great efforts to explore the possible wetting behaviors of water, oil, and gas^170^ on gel surfaces under various conditions, such as in air, underwater, or under organic liquids.

Bioinspired gel surfaces with superwettability and special adhesion hold potential to overcome the challenges faced in diverse applications. For instance, scalable fabrication of functional composite thin films with highly ordered structures^171^ challenges the traditional methods, e.g., interfacial polymerization at the oil/water interface.^172^ The superspreading of liquids on immersed gel surfaces provides a promising way to address such problem by utilizing the spreading‐induced shear forces and formed thin liquid layers. Although numerous efforts have been devoted to improving the performance of implants,^173^, ^174^, ^175^, ^176^ such as the blood vessel prosthesis, nearthrosis, and contact lens, none of them can compare favorably with natural ones due to the lack of some essential features, for example, biocompatibility, durability, and self‐repair ability.^177^, ^178^ In this case, gel‐based surfaces may be useful because their similar framework to the extracellular matrix of organs offers the opportunity to coat implants to minimize the chance of rejection.^179^, ^180^, ^181^ Therefore, numerous opportunities are available to settle critical issues in areas ranging from chemistry and materials science to biology. Along with these opportunities, we envision the development of new concepts and ideas that will reshape our understanding of bioinspired gel surfaces, which will significantly benefit our healthcare and daily life in the near future.

## Conflict of Interest

The authors declare no conflict of interest.
